# Evidence for the Contribution of the miR-206/BDNF Pathway in the Pathophysiology of Depression

**DOI:** 10.1093/ijnp/pyae039

**Published:** 2024-09-02

**Authors:** Ya-Bin Zheng, Xiang Jin

**Affiliations:** Department of Neurology, The Second Hospital of Nanjing, Nanjing University of Chinese Medicine, Nanjing, China; Department of Pharmacy, The Second People’s Hospital of Nantong, Nantong, China

**Keywords:** Depression, MiRNA, depressive-like behavior, neurogenesis, biomarker

## Abstract

Depression is a complex disorder with substantial impacts on individual health and has major public health implications. Depression results from complex interactions between genetic and environmental factors. Epigenetic mechanisms, including DNA methylation, microRNAs (miRNAs), and histone modifications, can produce heritable phenotypic changes without a change in DNA sequence and recently were proven to mediate lasting increases in the risk of depression following exposure to adverse life events. Of these, miRNAs are gaining attention for their role in the pathogenesis of many stress-associated mental disorders, including depression. One such miRNA is microRNA-206 (miR-206), which is a critical candidate for increasing the susceptibility to stress. Although miR-206 is thought to be a typical muscle-specific miRNA, it is expressed throughout the brain, particularly in the hippocampus and prefrontal cortex. Until now, only a few studies have been conducted on rodents to understand the role of miR-206 in stress-related abnormalities in neurogenesis. However, the precise underlying molecular mechanism of miR-206–mediated depression-like behaviors remains largely unknown. Here, we reviewed recent advances in the field of biomedical and clinical research on the role of miR-206 in the pathogenesis of depression from studies using different tissues and various experimental designs and described how abnormalities in miR-206 expression in these tissues can affect neuronal functions. Moreover, we focused on studies investigating the brain-derived neurotrophic factor (BDNF) as a functional target of miR-206, where miR-206 has been implicated in the pathogenesis of depression by suppressing the expression of the BDNF. In summary, these studies confirm the existence of a tight correlation between the pathogenesis of depression and the miR-206/BDNF pathway.

## INTRODUCTION

Depression is a common psychiatric disorder that causes considerable pain and burden to patients and their families. According to the World Health Organization, depression is a substantial global public health concern, and more than 300 million people worldwide suffer from this disorder, making it the leading cause of disability (Mathers and Loncar, 2006). It is characterized by changes in emotional state, such as depressed mood, social isolation, and anhedonia, as well as cognitive impairments, such as concentration and memory difficulties (Dehn and Beblo, 2019). In addition to its primary effects, depression also causes secondary disability, because patients with depression are less likely to comply with medical treatment and are highly susceptible to developing other chronic medical illnesses (Saraceno, 2005). Depression can be divided into mild, moderate, and major depressive disorder (MDD) according to the type and severity of symptoms. Based on the time of onset in a specific population, depression can be divided into child, postpartum, menopausal, and senile depression. Depression has serious negative social influence; for example, approximately 5% of the individuals in the United States had serious thoughts of suicide in 2019, and over 30% of the individuals suffering from MDD reported suicidal ideation (Nikayin and Sanacora, 2021).

Depression is etiologically complex, and environmental stress is widely recognized as one of its main risk factors. Genetic factors also play a role in depression etiology. In-depth studies of the pathophysiological mechanisms underlying depression have recently identified several mechanisms, including deficiencies in monoamine neurotransmission, vascular changes, increased inflammation, hypothalamic-pituitary-adrenal axis abnormalities, and reduced neurogenesis and neuroplasticity (Dean and Keshavan, 2017). Moreover, meta-analytic evidence using neuroimaging modalities indicates that volumetric reductions in the hippocampus, anterior cingulate cortex, PFC, striatum, and amygdala are frequently observed in depressed adults (Kempton et al., 2011). Antidepressants need to be seen as part of a package of treatment for the patient with depression, which also includes psychological treatments and social interventions, while psychotherapy, exercise therapy, and electroconvulsive therapy may also be effective (Kok and Reynolds, 2017). However, not all individuals respond to these treatments; 1 in 3 adults with MDD does not experience clinically significant improvement after multiple sequential courses of antidepressants and has treatment-resistant depression (Rush et al., 2006). In addition, many depressive disorders are not recognized, are misdiagnosed, or are mistreated by primary care physicians. Although there are many basic and clinical studies on depression, the pathogenesis underlying its development remains unclear, which limits the development of diagnostic and therapeutic approaches for this disorder.

The biological impact of environmental factors in depression and other stress-related disorders is mediated by a variety of epigenetic modifications. Alterations of these epigenetic modifications may contribute to neurotransmission and neuroglia dysfunction, neuroplasticity impairment, and abnormal neuroendocrine responses, which are involved in the pathophysiology of depression (Otte et al., 2016; Malhi and Mann, 2018). Among epigenetic mechanisms, miRNAs may be of particular significance according to recent studies and have been proposed as epigenetic mechanisms mediating the long-lasting effects of stress (Alegría-Torres et al., 2011; Fan et al., 2022). MiRNAs are small noncoding RNA molecules (21–24 nucleotides), recognized to destine the targeted mRNA for degradation or translational inhibition via binding to the 3ʹ-untranslated regions (UTRs) of numerous target gene mRNAs by a short “seed sequence” in a semi-complementary manner (Bartel, 2004). MiRNAs are also responsible for the sequence-specific and post-transcriptional regulation of the expression of more than 60% of the genes in mammals (Friedman et al., 2009), and aberrant expression of miRNAs is an important factor in the development and progression of diseases, including depression. For example, compared with those of healthy controls, 2 miRNAs in the plasma exosomes of patients with treatment-resistant depression showed significant differences in expression (Li et al., 2021a). Studies have also focused on miRNAs in the brain tissue of patients with depression. For example, compared with those of nonpsychiatric controls, a lower expression of 21 miRNAs has been observed in the PFC of antidepressant-free depressed suicide subjects (Smalheiser et al., 2012). Thus, these findings highlight the potential of miRNAs as biomarkers for the treatment response in depression.

MiR-206, a member of the miR-1 family, is one of the most-studied and well-characterized miRNAs. It is specifically expressed in skeletal muscles and has been shown to be involved in the pathogenic processes of a variety of human disorders, including cancer (Wang et al., 2019), amyotrophic lateral sclerosis (Toivonen et al., 2014), and Alzheimer disease (Lee et al., 2012). A recent study reported that miR-206 could function as a feasible antidepressant target for depression (Guan et al., 2021). However, the exact mechanisms underlying the role of miR-206 in depression remain unclear. In this review, we summarize the role of miR-206 in the pathogenesis of depression and discuss its potential therapeutic implications. This evidence will improve our understanding of the clinical significance of miR-206 as a biomarker in depression.

## BIOLOGICAL CHARACTERISTICS OF miR-206

### Chromosomal Localization and Expression Patterns of miR-206

As a member of the miR-1 family, miR-206 is located between interleukin-17 and polycystic kidney and hepatic disease 1 (*PKHD1*) genes on chromosomes (chr) 6p12.2 in humans, chr 1 in mice, and chr 9 in rats (Ma et al., 2015). miR-206, a vertebrate-specific miRNA, is highly conserved in terms of its genomic organization and sequence. It is considered a myomiR and not only contributes to the regeneration of muscles during injury but also promotes myogenic differentiation (Anderson et al., 2006). Sempere et al. showed the first description of miR-206 specifically expressed in striated muscles and demonstrated that miR-206 was expressed in mouse and human skeletal muscle and heart, with low expression in other organs (Sempere et al., 2004). During mouse embryonic development, miR-206 was expressed in skeletal myoblasts in head, limb buds, and somites muscles. A very low expression of miR-206 was first detected in mice at 9.5 days post coitum, and then, the level of miR-206 started to significantly increase around 11.5–12.5 days post coitum (Takada et al., 2006). New findings show miR-206 has regulatory roles in the development of other mammalian nonmuscle tissues, including nerve, brain structure, adipose, and some specialized immunological cells (Mitchelson and Qin, 2015). MiR-206 was significantly expressed in various brain regions correlated with depression such as the hippocampus, medial prefrontal cortex (mPFC), hypothalamus, amygdala, nucleus accumbens (NAc), and ventral tegmental area (VTA) (Guan et al., 2021). Research indicated that chronic social defeat stress (CSDS) exposure increased the level of miR-206-3p in the hippocampus and mPFC of mice while downregulating the level of miR-206-3p in the VTA. In contrast, CSDS did not influence the miR-206-3p level in the hypothalamus, amygdala, and NAc of mice (Guan et al., 2021). Recently, an increasing number of preclinical studies have shown that miR-206 is upregulated in the hippocampal tissue, cerebrospinal fluid, and plasma, and that increased miR-206 downregulates the expression of BDNF (Tian et al., 2014; Wang et al., 2017; Shao and Xu, 2022).

### Biogenesis of miR-206

**Figure 1.**
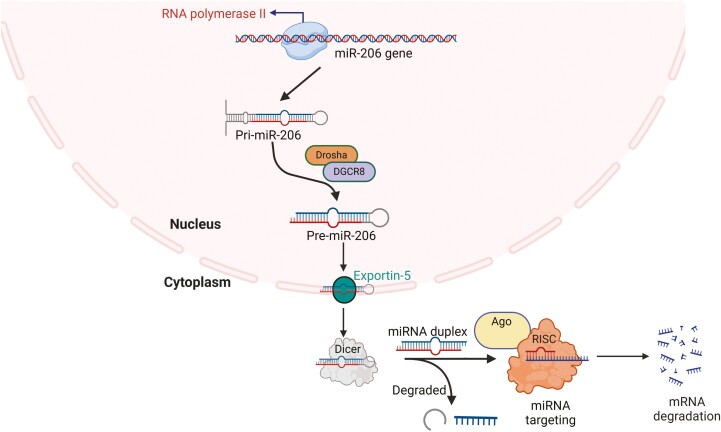
miR-206 biogenesis. The biogenesis of miR-206 is initiated by RNA polymerase II, which transcribes miR-206 genes as pri-miR-206. Pri-miR-206 is then trimmed in the nucleus into shorter stem-loop secondary structures known as pre-miR-206 with the Drosha and DGCR8. Henceforth, the exportin-5 protein transports the pre-miR-206 from the nucleus to the cytoplasm, and the Dicer processes the pre-miR-206 into a form of short double-stranded miRNA duplex. Next, the duplex is loaded into the RISC and separated into 2 strands: 1 strand is degraded, whereas the other associates with an AGO protein within the RISC, binds to the target RNAs, and cleaves them. The figure was generated using BioRender (Agreement number: CU27AY7PTZ).

MiR-206 biogenesis follows a programmed pathway to produce a mature effector molecule mediated by 2 ribonuclease III enzymes: Drosha and Dicer (O’Brien et al., 2018). Specifically, the biogenesis of miR-206 is initiated by RNA polymerase II, which transcribes miR-206 genes into long, capped, polyadenylated RNA molecules named primary miR-206 (pri-miR-206). Pri-miR-206 is then trimmed in the nucleus into shorter (60–100 nt) stem-loop secondary structures, known as precursor miR-206 (pre-miR-206), with RNA endonuclease III (Drosha) and its binding protein (DGCR8/Pasha). DGCR8/Pasha, a double-stranded RNA-binding domain protein, recognizes stem-loop structures in pri-miR-206, whereas Drosha cleaves pre-miR-206 hairpins from primary transcripts (pri-miR-206). Subsequently, the exportin-5 protein transports pre-miR-206 from the nucleus to the cytoplasm (Bohnsack et al., 2004), and the ribonuclease III endonuclease, Dicer, processes pre-miR-206 into a short double-stranded miRNA duplex (18–25 nucleotides) (Yi et al., 2003), which is then processed into a mature miRNA. Next, the duplex is loaded into the RNA-induced silencing complex (RISC) and separated into 2 strands. One strand is degraded, whereas the other associates with an argonaute (AGO) protein within the RISC and binds to the target RNAs to cleave them (Figure 1) (Yi et al., 2003).

### Research Progress Related to Upstream Regulatory Targets of miR-206

Ju and Yang provided the role and mechanism of long noncoding RNA H19 (H19) in regulating temporal lobe epilepsy (Ju and Yang, 2022). They found the level of H19 was upregulated and miR-206 expression was downregulated in rat hippocampus neurons after kainic acid (KA) treatment, while both H19 knockdown and miR-206 overexpression decreased KA-induced oxidative stress, inflammatory response, hippocampus neuronal apoptosis, and autophagy (Ju and Yang, 2022). The authors concluded that H19 knockdown reduced KA-induced hippocampus neuron injury, presumably through directly upregulating miR-206 and activating the phosphatidylinositol 3-kinase/protein kinase B (AKT) signaling pathway (Ju and Yang, 2022). Another study by Lee et al. demonstrated that ionizing radiation induced cognitive impairment in mice through upregulating miR-206-3p targeting p21-activated kinase 3 (PAK3), leading to the downregulation of PAK3-LIM kinase 1-cofilin signaling (Lee et al., 2023), while intranasal administration of antagomiR-206-3p recovered PAK3 levels and cognitive function in irradiated mouse models. These findings indicate intranasal administration of antagomiR-206-3p may offer a promising approach for reducing unexpected side effects of cranial irradiation.

### Prediction of miR-206 Target Genes

**Figure 2.**
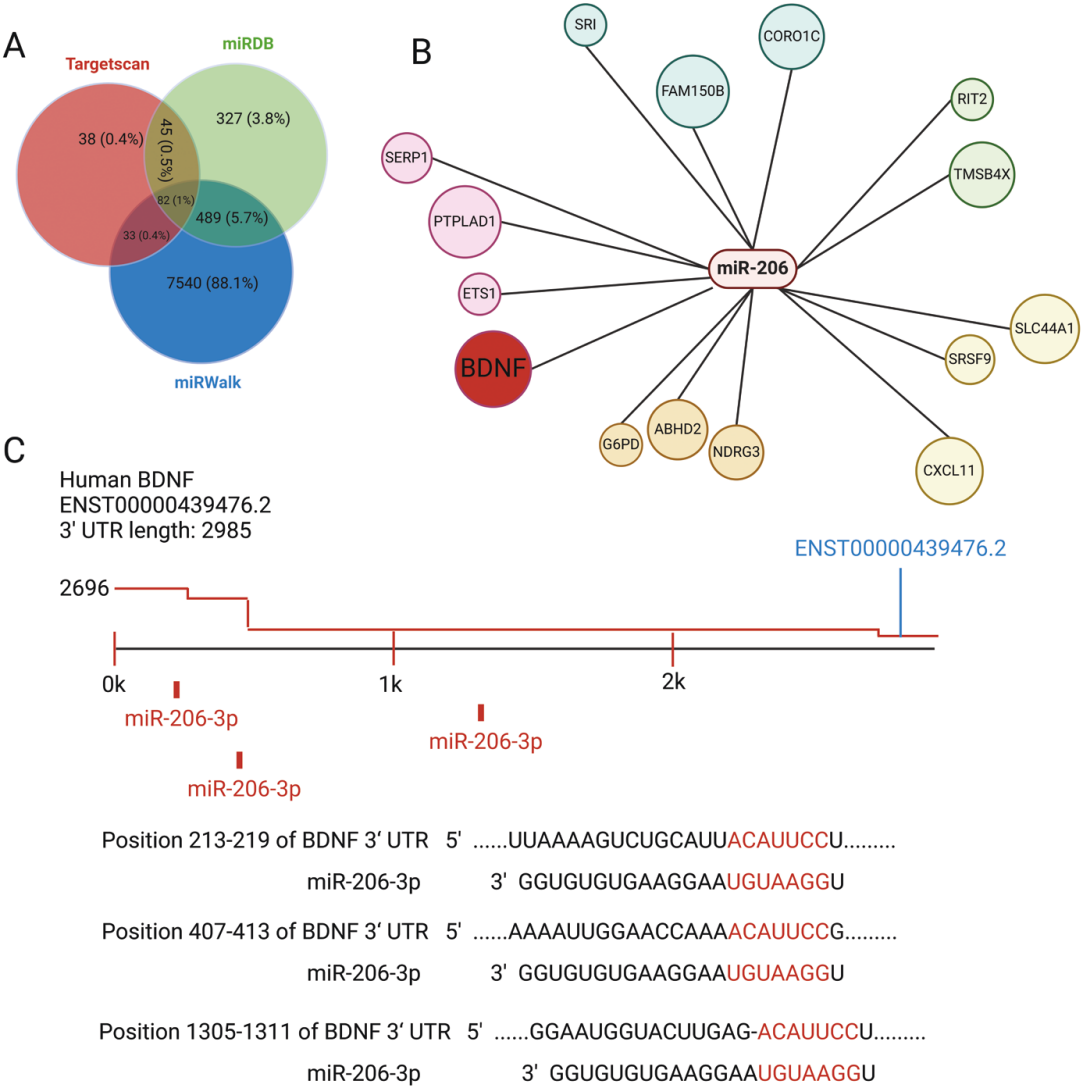
miR-206 target genes. (A) A prediction of a total of 82 common target genes in 3 databases: TargetScan, miRDB, and miRWalk. The left panel was adapted from Khalilian et al. (Khalilian et al., 2022). (B) Bioinformatical prediction of 15 target genes of miR-206; BDNF appears to be the most likely related with neuronal regulation pathways. (C) Putative seed-matching sites between miR-206 and BDNF (Agarwal et al., 2015).

Several databases, including TargetScan (https://www.targetscan.org/), miRDB (http://www.mirdb.org/), and miRWalk (http://mirwalk.umm.uni-heidelberg.de/), were used to predict the miR-206 target genes. Using these 3 databases, 82 mRNAs were identified as common targets of this miRNA (Figure 2A). According to data from miRDB and TargetScan, miR-206 regulates many target genes, including the C-C Motif Chemokine Ligand 2 (Wu et al., 2019), Forkhead Box Protein 1 (Du et al., 2018), orthodenticle homeobox 2 (Panwalkar et al., 2015), ubiquitin specific peptidase 22 (Xie et al., 2023), Cbp/p300 interacting transactivator with Glu/Asp rich carboxy-terminal domain 2/serine/threonine kinase 39 (Li et al., 2023), brain-derived neurotrophic factor (BDNF), and so on (Figure 2B). BDNF is a protein that is largely found in certain brain regions, such as the hippocampus, cerebral cortex, and hypothalamus. BDNF is associated with neuronal growth and increased neuroplasticity, whereas reduced BDNF levels have been implicated in depression (Angelucci et al., 2005). Moreover, results of the dual-luciferase reporter assay from Sun et al. demonstrated that binding sites exist between BDNF and miR-206 (Figure 2C) (Sun et al., 2017). A growing body of research has recently suggested that miR-206 regulates the expression of BDNF, indicating that the inhibition of miR-206 increases the level of BDNF (Tapocik et al., 2014; Wang et al., 2014). Therefore, we considered that BDNF is a direct target of miR-206 in neurones and that miR-206 could regulate BDNF expression to participate in the development of depression.

## miR-206 as a Biomarker of Depression

A growing body of research now shows that miR-206 is involved in the pathogenesis of a variety of malignant and nonmalignant conditions (Qi and Guan, 2024). The dysregulation of miR-206 has been linked to many disorders in skeletal muscle such as Duchenne muscular dystrophy and amyotrophic lateral sclerosis (Ma et al., 2015). In the context of cancer, many studies have led to the supposition of miR-206 as a tumor suppressor miRNA (Bahari Khasraghi et al., 2023), although some exceptions have been demonstrated (Parasramka et al., 2012; Zhou et al., 2019). In recent years, studies have shown that miR-206 is involved in the pathophysiology of several neurodegenerative and neuropsychiatric disorders, including Alzheimer disease (Tian et al., 2014), depression (Guan et al., 2021), anxiety (Miao et al., 2018), and bipolar disorder type I (BD-I) (Wang et al., 2014). Based on bioinformatics analysis and other related researches, miR-206 is an miRNA that could target the important BDNF signal molecule (Shao and Xu, 2022). BDNF is a neurotrophin that is broadly expressed in the developing and adult mammalian brain and has been implicated in synaptic plasticity and neurogenesis (Yu and Chen, 2011). Therefore, miR-206 may show remarkable potential in the treatment of mood disorders and specifically in the treatment of clinical depression. Although scientists have gained a deeper understanding of the pathophysiology of depression over the past few decades, these advances in the understanding of molecular mechanisms have not translated into improved treatment outcomes because no validated biomarkers are available for depression. Therefore, there is an urgent need to identify diagnostic and prognostic biomarkers to develop a wider spectrum of novel therapeutics for depression. It is encouraging to see that miR-206 has recently emerged as an important mediator in the pathophysiology of depression and has the potential to be used as a target for antidepressants.

Several preclinical and clinical studies have repeatedly shown that stress and increased corticosterone levels are causally associated with depression-like phenotypes (McEwen, 2005; Pariante and Lightman, 2008). In a recent study, we reported that the level of plasma corticosterone was significantly increased in CSDS-induced mice, whereas the use of AAV-siR-206-3p abolished the effects of CSDS on plasma corticosterone levels in mice (Guan et al., 2021). This was also supported by a recent study by Yang et al., which reported altered expression levels of miR-206 in the serum exosomes of patients with traumatic brain injury (TBI) (Yang et al., 2024). TBI is associated with several psychiatric and neurobehavioral problems, including depression. TBI leads to remarkable neuropathological effects, including parenchymal loss in different brain areas, white matter disruption through diffuse axonal injury and neurochemical changes associated with a higher risk of developing depression after TBI (Armstrong et al., 2016; Smith et al., 2019; Mayer and Quinn, 2022). Although the individual mechanisms underlying depression and TBI have been widely studied, the neurobiological basis of depression following TBI remains largely unknown. It was found that the expression level of serum exosomal miR-206 showed an upward trend in the TBI group compared with that in the control group. In addition, further correlation analysis showed that the expression of serum exosomal miR-206 was positively correlated with the expression of BDNF, and a reduced level of serum exosomal miR-206, as a biological marker, showed good predictive value in patients with TBI (Yang et al., 2024). Although this study demonstrated that serum exosomal miR-206 is a novel biomarker for TBI, the downstream signaling molecules were not confirmed, and further research is needed to understand the precise mechanism. Similarly, He et al. reported that miR-206 levels were higher in the plasma of patients with moderate-to-severe stroke than those in patients with mild stroke (He et al., 2019), whereas no significant difference was detected in the effect of miR-206 on the diagnostic accuracy between patients with favorable and unfavorable outcomes. Post-stroke depression is the most frequent psychiatric complication of stroke, and clinical studies have revealed that lesions in the left hemisphere tend to be associated with a higher incidence of depression (Robinson et al., 1984). We speculate that this contradictory result may be attributed to diverse clinical conditions, distinct sample collection times, and heterogeneity among the different populations. Therefore, in future studies, the sample size should be expanded. In comparison, after an exploratory analysis using the online miRNA analysis software FunRich, Wu et al. focused on the levels of miR-206 in the plasma of patients with MMD and healthy controls (Wu et al., 2022). It was found that the level of hsa-miR-206 was downregulated in MMD and that it could mediate the onset and development of MMD by governing the immune-inflammatory response and neuronal remodeling Wu et al. (2022). Although the above study provides some theoretical underpinnings of nerve-immunity interaction treatments for the brains of patients with MMD, it has several limitations. As a result, more scientific and clinical research studies are required to confirm the above-mentioned findings.

## MiR-206 IN PATHOGENESIS OF DEPRESSION

### MiR-206 Was Implicated in Pathogenesis of Depression by Suppressing BDNF Expression

**Figure 3.**
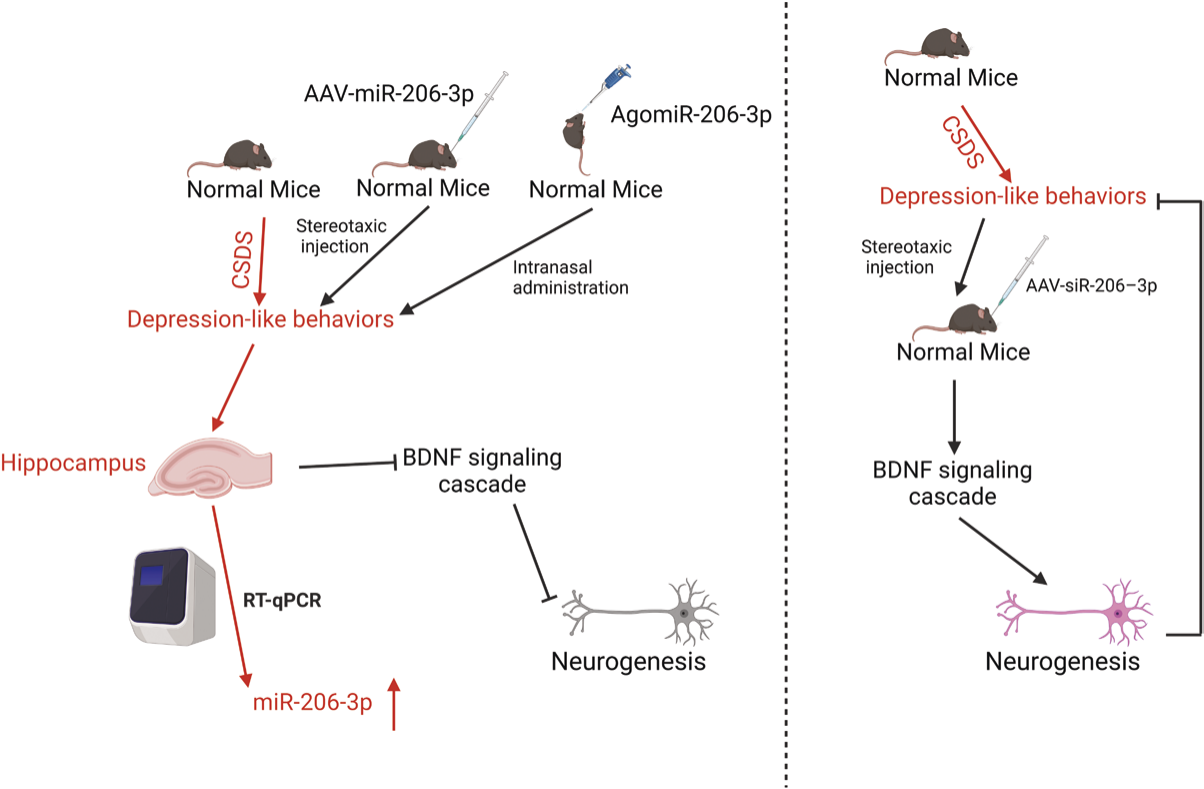
miR-206-3p expression was significantly increased in the hippocampus of CSDS-induced mice by quantitative real-time reverse transcription PCR (qRT-PCR) compared with that of normal mice Guan et al. (2021). In addition, mice with increased miR-206-3p activity were more prone to develop depression-like behaviors, whereas those with decreased miR-206-3p activity were more stress resilient. Moreover, the overexpression of miR-206-3p in the hippocampus of mice induced notable depressive-like effects by negatively regulating the expression of BDNF signaling cascade and the amount of doublecortin (DCX) cells, whereas the inhibition of miR-206-3p alleviated the depressive symptoms of stressed mice through the upregulation of BDNF signaling and neurogenesis Guan et al. (2021). The figure was generated using BioRender (Agreement number: HP27AY7S7P).

Evidence from cell lines and animal models has revealed the essential role of miR-206 in animal adaptation to environmental stress (Solomon et al., 2019), which has important implications for the study of the role and molecular mechanisms of miR-206 in the pathogenesis of human depression. A wealth of data show that miR-206 plays an essential role in neuropsychiatric disorders, such as depression (Chang et al., 2020). Depression is characterized by impairment in adult neurogenesis, while altered adult hippocampal neurogenesis is crucial for the development and treatment of depression. A recent study by our laboratory made a substantial contribution to our understanding of the role of miR-206 in the pathogenesis of depression (Figure 3) (Guan et al., 2021). We found that miR-206-3p (a form of miR-206) was significantly increased in the hippocampus of mice subjected to CSDS model, suggesting that elevated levels of miR-206-3p in the hippocampus correlate with chronic stress (Guan et al., 2021). Stereotaxic intracranial injection with a lentiviral vector or intranasal administration of an miR-206-3p agonist for miR-206-3p overexpression was also employed to validate the biological mechanism. Mice with increased miR-206-3p activity were more prone to developing depression-like behaviors, whereas those with decreased miR-206-3p activity were more stress resilient (Guan et al., 2021). Furthermore, the overexpression of miR-206-3p in the hippocampus of mice induced notable depressive-like effects by negatively regulating the expression of the BDNF signaling cascade and the number of doublecortin cells, whereas the inhibition of miR-206-3p alleviated depressive symptoms in stressed mice through the upregulation of BDNF signaling and neurogenesis (Guan et al., 2021). In summary, miR-206–mediated downregulation of BDNF expression is a novel target for the development of depression. Remarkably, CSDS exposure increased the level of miR-206-3p in the hippocampus but did not affect the level of miR-206-5p in any other brain regions (Guan et al., 2021). This implies that miR-206-3p expression in the hippocampus is mostly correlated with chronic stress, which is partially consistent with previous reports (Li et al., 2023). Therefore, further studies are required to clarify the role of miR-206-5p in depression and the target genes and pathways involved.

**Figure 4.**
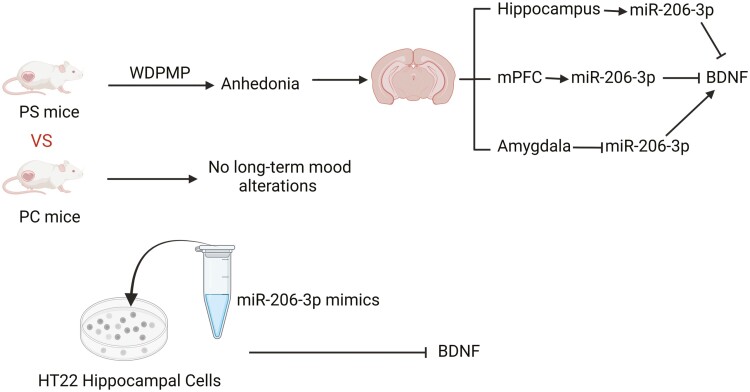
Decreased BDNF expression in both the hippocampus and mPFC of pregnant stressed (PS) mice was found to be associated with enhanced miR-206-3p levels, which would further lead to transient anhedonia in pregnant mice. In addition, it was observed that the expression levels of particular *Bdnf* variants and BDNF protein were significantly decreased in the miR-206 mimic group compared with those of the NC group after transfecting miR-206-3p mimics into HT22 hippocampal cells. These behaviors were associated with decreased BDNF expression and increased miR-206-3p expression in the hippocampus and mPFC, as well as increased BDNF expression and decreased miR-206-3p expression in the amygdala of PS mice (Miao et al., 2018). The figure was generated using BioRender (Agreement number: PB27AY7WSH).

Similarly, another study found that decreased BDNF expression in both the hippocampus and mPFC of pregnant stressed mice was associated with enhanced miR-206-3p levels (Miao et al., 2018), which further led to depression/anxiety-related behaviors in pregnant mice (Figure 4). During the study, miR-206-3p mimics were transfected into HT22 hippocampal cells, and it was observed that the expression levels of particular *Bdnf* variants and BDNF protein were significantly decreased in the miR-206 mimic group compared with those in the normal control group (Miao et al., 2018), suggesting that miR-206-3p negatively regulates BDNF expression. Moreover, miR-206-3p expression increased in the hippocampus and mPFC, whereas decreased miR-206-3p levels were observed in the amygdala of PS mice (Miao et al., 2018). This result is inconsistent with our findings (Guan et al., 2021). We speculate that this contradictory finding might be attributed to differences in animal models. Overall, considerable variation was observed between studies, and further work is warranted.

In contrast, some studies have not found a statistically significant association between individual polymorphisms (miR-206 rs16882131 or BDNF rs6265) and the risk of BD-I or treatment response (Wang et al., 2014). However, the potential interplay between miR-206 rs16882131 and BDNF rs6265 contributes to an increased risk of BD-I and a poorer treatment response (Wang et al., 2014). This finding supports previous reports in which miR-206 was implicated in the pathogenesis of depression by suppressing BDNF expression (Guan et al., 2021). Thus, the effect of miR-206 rs16882131 on the susceptibility to BD-I and treatment response might act through the regulation of BDNF expression. Therefore, a larger sample size would be required to investigate this possibility.

### miR-206 and Its Regulation of BDNF in Neural Circuit Function after Brain Exposure to Alcohol

Recent clinical and preclinical evidence suggests that chronic ethanol exposure is associated with an increased risk of depressive disorders (Yao et al., 2021), but the underlying mechanism is not clear. A recent study showed that chronic intermittent ethanol (CIE) and forced swim stress (FSS) exposure reduced the mRNA expression of BDNF, whereas miR-206 levels were increased in the mPFC, central amygdala and hippocampus of adult male C57BL/B6J mice by quantitative polymerase chain reaction assay (Solomon et al., 2019). These results strongly suggest that CIE exposure induces elevated miR-206 expression, thereby reducing BDNF expression in several brain regions, which is consistent with most findings (Miao et al., 2018; Guan et al., 2021). Nevertheless, acute and repeated FSS (5 daily 10-minute FSS sessions) alone and in combination with a history of CIE exposure also induced a significant increase in miR-206 levels in the mPFC and central amygdala compared with those of controls (Solomon et al., 2019). However, these changes appeared to normalize in the CIE and FSS groups at this time point in the hippocampus. We consider that the expression levels of miR-206 are altered in a brain region–specific and time-dependent manner. Further investigations are required to explain these differences.

Consistent with this, Tapocik et al. found that the level of miR-206 was upregulated in the mPFC of rats 3 weeks after completion of alcohol exposure, whereas it was absent in VTA, NAc, and amygdala (Tapocik et al., 2014), indicating that the upregulation of miR-206 in the brain region of alcohol-vapor-exposed rats was also regionally selective. The same study also investigated the association between upregulated miR-206 and alcohol preference and discovered that miR-206 overexpression in the mPFC induced escalated alcohol self-administration (Tapocik et al., 2014). Furthermore, it demonstrated that miR-206 binds to and inhibits the expression of the BDNF transcript in HEK293T cells by measuring luciferase activity and protein levels in the mPFC of rats as well as the secretion of BDNF protein in primary rat cortical neurones (Tapocik et al., 2014). Therefore, chronic brain alcohol exposure might increase miR-206 expression and lead to a decrease in BDNF expression within the mPFC, thereby impairing synaptic plasticity in the mPFC and ultimately contributing to the onset of depression. This result is in agreement with those of previous studies and supports the notion that miR-206–mediated impairment in synaptic plasticity may underlie the behavioral consequences of depression (Guan et al., 2021). However, it was found that the levels of BDNF were elevated in the NAc following alcohol dependence (Tapocik et al., 2014). One possible interpretation is that BDNF acts as a stress-susceptible molecule in the NAc (Pagliusi et al., 2022). The NAc is a target of the mesolimbic dopamine system, which receives dopamine inputs from dopaminergic neurones in the midbrain (Ikemoto, 2007). Additionally, CSDS increases BDNF levels in the NAc of susceptible mice that display social avoidance and anhedonia (Berton et al., 2006; Pagliusi et al., 2018), whereas chronic antidepressant treatment can reverse these pathological changes (Jiang et al., 2014). These results imply that BDNF plays opposite roles in the VTA-NAc and hippocampal-prefrontal circuits.

### Role of Gut Microbiota Regulating miR-206 Expression in Depression

**Figure 5.**
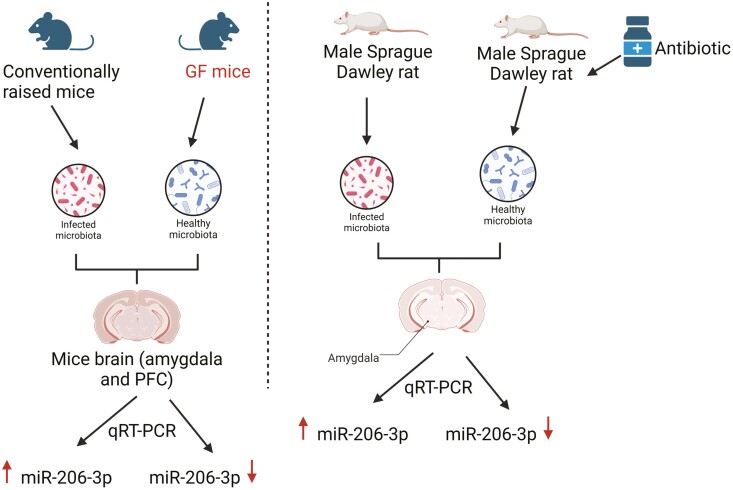
miR-206-3p showed a relatively large decrease in both the amygdala and PFC of GF mice by qRT-PCR validations, whereas a significant decrease in miR-206-3p was observed in the amygdala of rats exposed to antibiotics, which was in line with the data from GF mice (Hoban et al., 2017). The figure was generated using BioRender (Agreement number: QS27AY7ZLI).

Accumulating evidence supports the view that abnormal gut microbiota plays an important role in the pathogenesis of depression, and correcting these disturbances could alleviate depression (Winter et al., 2018). Because of the important roles of miRNAs in gene regulation at both the post-transcriptional and translational levels, the importance of miRNAs in host-microbiota interactions and their impact on many psychiatric disorders have also begun to be appreciated. Based on the evidence mentioned above, miR-206 has been implicated in depression-like behaviors. However, it remains unknown whether miR-206 is recruited by the gut microbiome to participate in the pathogenesis of depression. Some of the first investigations into the microbial regulation of miR-206 expression were conducted on germ-free (GF) rodents (Figure 5) (Hoban et al., 2017). Authors found miR-206-3p showed a relatively large decrease in both the amygdala and PFC of GF mice by quantitative reverse transcriptase PCR (qRT-PCR) validation(Hoban et al., 2017). Moreover, a significant decrease in miR-206-3p was observed in the amygdala of rats exposed to antibiotics (Hoban et al., 2017), which is in line with the data from GF mice (Hoban et al., 2017). These results indicate that the regulation of miR-206-3p expression within the amygdala of animals is influenced by microbiota composition, which helps us to better understand how the gut microbiota affects the development of depression. However, the authors did not investigate the effect of gut microbiota on BDNF expression, and further studies should be conducted to investigate the precise mechanism underlying the regulation of gut microbiota-mediated miR-206-3p expression in depression.

## ROLE OF miR-206 IN THE ANTIDEPRESSANT EFFECT OF KETAMINE

For further exploring the potential therapeutic targets of miR-206 in the treatment of clinical depression, we also reviewed several studies and found miR-206 was involved in novel therapeutic targets for the anti-depressive effect of ketamine (Yang et al., 2014).

Previous investigations have shown that ketamine, an N-methyl-D-aspartate receptor antagonist, exerts fast and long-lasting antidepressant effects (Berman et al., 2000). It is reported that ketamine can be used to treat MDD through single or repeated infusions (Murrough et al., 2013). According to the hypothesis of ketamine’s disinhibition, it can selectively block N-methyl-D-aspartate receptors expressed in gamma-aminobutyric acid (GABA)-inhibitory interneurons, reduce the GABA release, and then reduce the inhibition of glutamate neurons (Grunze et al., 1996; Zorumski et al., 2016). Alternatively, the main effects of ketamine on depression mainly rely on the activation of postsynaptic alpha-amino-3-hydroxy-5-methyl-4-isoxazolepropionic acid (AMPA) receptors (Autry et al., 2011) and the subsequent activation of downstream neuroplasticity-related signaling pathways, including the upregulation of mechanistic target of rapamycin (mTOR) and BDNF (Zhou et al., 2014), thereby enhancing the efficacy of synaptic transmission that are involved in the behavioral antidepressant actions of ketamine. Therefore, the antidepressant-like effects of AMPA activation and NMDA inhibition complement each other. However, the adverse effects associated with ketamine treatment, particularly its dissociative properties, limit its broad application. These limitations have led investigators to explore the exact mechanisms of action underlying the antidepressant clinical responses of ketamine to develop novel rapid-acting antidepressants with fewer undesirable side effects.

**Figure 6.**
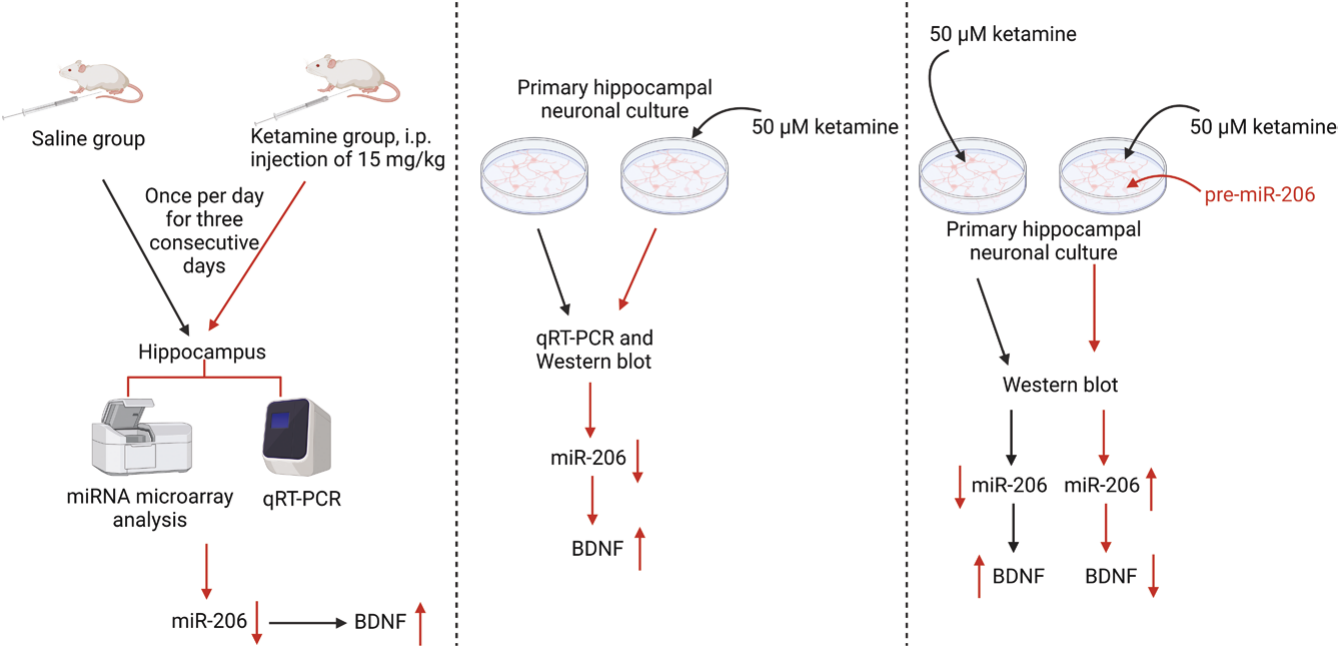
miR-206 expression signal intensity was downregulated after 3-day ketamine injection compared with that of saline-treated rats by the miRNA microarray analysis. Besides, miR-206 was decreased by approximately 50% in the hippocampus of ketamine-treated rats by using the qRT-PCR technique. In line, this result was observed in cultured hippocampal neurones after 50-µM ketamine treatment. In the same study, the expression of BDNF was significantly increased in the hippocampus of rats with ketamine treatment, whereas miR-206 overexpression (pre-miR-206) substantially reduced the BDNF expression induced by ketamine in the primary hippocampal neuronal cultured cells (Yang et al., 2014). The figure was generated using BioRender (Agreement number: CT27AY7YIP).

In line with these observations, Yang et al. reported that miR-206 was downregulated in ketamine-treated rats (Figure 6) (Yang et al., 2014). In this study, miRNA microarray analysis revealed that the miR-206 expression signal intensity was downregulated after 3-day ketamine injection compared with that of saline-treated rats. To verify this discovery, Yang et al. using the qRT-PCR technique to find that miR-206 was decreased by approximately 50% in the hippocampus of rats treated with ketamine (Yang et al., 2014). Furthermore, this result was also observed in cultured hippocampal neurones after 50 µM ketamine treatment (Yang et al., 2014). Equally important, the authors reported miR-206 overexpression (pre-miR-206) substantially reduced the BDNF expression induced by ketamine in primary hippocampal neuronal cultured cells, while BDNF expression was significantly increased in the hippocampus of rats with ketamine treatment (Yang et al., 2014), indicating that BDNF expression was strongly repressed by miR-206. These results suggest that miR-206/BDNF cascade is a novel therapeutic target for the antidepressant effects of ketamine. However, as shown in the above studies, the antidepressant effects of ketamine are associated with AMPA receptor-mediated upregulation of mTOR and BDNF (Xu et al., 2013; Zhou et al., 2014). Hence, we believe that miR-206 may modulate the expression of mTOR. Moreover, growing evidence indicates a role of miRNAs in ketamine-induced neurotoxicity (Fan et al., 2021; Yang and Long, 2024). Thus, miR-206 overexpression not only reduced the BDNF expression induced by ketamine but also led to ketamine-induced neurotoxicity, which then blocked the antidepressant actions of ketamine. Although these studies suggest that miR-206 is a critical novel gene involved in ketamine-induced BDNF expression, further studies are needed to understand the role of miR-206 in the antidepressant effects of ketamine.

## CONCLUSIONS

miRNAs are small endogenous RNAs that post-transcriptionally regulate gene expression. Recent studies have shown that miRNAs clearly play a role in the development and pathophysiology of depression (Li et al., 2020, 2021b). miR-206, a member of the miR-1 family, participates in the pathogenesis of various malignant and nonmalignant conditions (Zhou et al., 2013; Moon et al., 2016). To date, most studies have focused on the role of miR-206 in suppressing the growth of multiple cancer cells (Liao and Peng, 2020; Liu et al., 2020). However, miR-206 may play a central role in the pathophysiology of depression. In this review, we summarize and discuss the recent advances in preclinical and clinical research on the role of miR-206–mediated regulation of gene expression in the development of depression.

Several studies showed BDNF expression was repressed by miR-206 in a 3’-UTR reporter assay, confirming BDNF as a functional target of miR-206 (Miura et al., 2012; Sun et al., 2023). As an important neurotrophin, BDNF is synthesized in the brain and is crucial for neuronal survival, differentiation, and development (Martinowich and Lu, 2008). Available literature indicates that BDNF is involved in the pathophysiology of depression and antidepressant responses (Castrén and Rantamäki, 2010). Our previous research showed that miR-206-3p overexpression induced depressive-like behaviors in mice by downregulating BDNF expression, while knockdown of miR-206-3p in the hippocampus produced significant antidepressant-like effects and prevented decreased BDNF level in the CSDS model of depression (Guan et al., 2021), suggesting that miR-206 regulation of BDNF in the hippocampus may contribute to depression-like behaviors that emerge following chronic stress. Recent reports support these findings and show that the BDNF-mTORC1 signaling pathway is a crucial target for alleviating depressive-like behaviors and mediating the rapid antidepressant-like effects of various antidepressants (Duman, 2014; Wohleb et al., 2016, 2017). Based on these findings, we have reason to believe that miR-206 overexpression directly targets BDNF and represses the activation of the downstream mTOR signaling pathway. Therefore, further investigations are required to elucidate the precise mechanism of action of miR-206 in depression.

Recent studies have suggested that miR-206 mediates the rapid antidepressant-like effects of ketamine (Yang et al., 2014). Ketamine, an NMDA receptor antagonist, produces a rapid antidepressant action by blocking NMDA receptors in inhibitory neurones, which improves extracellular glutamate levels and activates AMPA receptors. This in turn contributes to the release of BDNF and activates the mTORC1 pathway, which promotes the growth of neurones and synapses and plays an antidepressant-like role. Thus, high expression of miR-206 could attenuate the increased level of BDNF induced by ketamine, which ultimately reduces the rapid-acting antidepressant effects of ketamine and contributes to the development of depression. Despite the promise of ketamine, key challenges including how to maintain response, concerns regarding short and long-term side effects and the potential for abuse remain. The most common side effects of ketamine noted in clinical studies include psychedelic symptoms (hallucinations, memory defects, panic attacks), nausea/vomiting, somnolence, and cardiovascular stimulation (Ceban et al., 2021). In addition to these transient and mild symptoms, there is considerable evidence that high doses of ketamine can lead to long-term cognitive impairment (Zhornitsky et al., 2022). With regard to the adverse effects of ketamine, an miR-206 antagonist may result in novel rapid-acting antidepressants with fewer undesirable side effects.

Collectively, these observations indicate that miR-206 binds to BDNF and inhibits its expression in depressive disorders. These results emphasize the importance of the BDNF signaling pathway in alleviating depressive behaviors. Based on these findings, miR-206/BDNF cascade may be a promising target for the treatment of depression.

### Limitations

This review has some limitations. First, most studies were conducted in animal models of depression, while there have been few scientific studies with humans. In addition, the number of animals used in experiments was small, and bioinformatics analysis may lead to weak statistical significance. More importantly, only male mice were used in most studies; thus, researchers will also include female mice in the research scope in the future. Furthermore, it should be noted that while miR-206 regulates target gene expression through binding the 3ʹ-UTR of the respective mRNA, it is known that miR-206 can bind the coding region or the 5ʹ-UTR of target genes. We did not focus our efforts on these regions as it requires a more complex bioinformatic analysis to identify these binding sites. Therefore, further studies on these important issues are needed.

## Data Availability

All the data extracted from included original articles are available in PubMed or Web of Science. This review was conducted without previous registration, and no protocol document was prepared.
